# Therapeutic administration of Tregitope-Human Albumin Fusion with Insulin Peptides to promote Antigen-Specific Adaptive Tolerance Induction

**DOI:** 10.1038/s41598-019-52331-1

**Published:** 2019-11-06

**Authors:** Anne S. De Groot, Gail Skowron, James Robert White, Christine Boyle, Guilhem Richard, David Serreze, William D. Martin

**Affiliations:** 1 EpiVax, Inc., 188 Valley St., Providence, RI 02909 USA; 2 Resphera Biosciences, Baltimore, MD 21231 USA; 3 Jackson Laboratories, Bar Harbor, ME 04609 USA

**Keywords:** Autoimmunity, Type 1 diabetes

## Abstract

Type 1 Diabetes (T1D) is an autoimmune disease that is associated with effector T cell (Teff) destruction of insulin-producing pancreatic beta-islet cells. Among the therapies being evaluated for T1D is the restoration of regulatory T cell (Treg) activity, specifically directed toward down-modulation of beta-islet antigen-specific T effector cells. This is also known as antigen-specific adaptive tolerance induction for T1D (T1D ASATI). Tregitopes (**T reg**ulatory cell ep**itopes**) are natural T cell epitopes derived from immunoglobulin G (IgG) that were identified in 2008 and have been evaluated in several autoimmune disease models. In the T1D ASATI studies presented here, Tregitope peptides were administered to non-obese diabetic (NOD) mice at the onset of diabetes within two clinically-relevant delivery systems (liposomes and in human serum albumin [HSA]-fusion products) in combination with preproinsulin (PPI) target antigen peptides. The combination of Tregitope-albumin fusions and PPI peptides reduced the incidence of severe diabetes and reversed mild diabetes, over 49 days of treatment and observation. Combining HSA-Tregitope fusions with PPI peptides is a promising ASATI approach for therapy of T1D.

## Introduction

Type 1 Diabetes (T1D) is an autoimmune disease that is associated with effector T cell (Teff) destruction of insulin-producing pancreatic beta-islet cells. In non-diabetics, these self-reactive T cells are deleted during thymic development, rendered anergic, or converted into natural regulatory T cells (Tregs) that suppress autoimmune responses. In T1D, for reasons that are not well understood, these regulatory mechanisms fail, with the resultant destruction of beta-islet cells, insulin deficiency, and glucose intolerance. Among the therapies being evaluated for T1D is the restoration of Treg activity, specifically directed toward down-modulation of beta-islet antigen-specific T effector cells.

Previous efforts to enhance Treg activity in T1D have included low-dose IL-2 in combination with immune-suppressant medications such as rapamycin^1^ and direct adoptive transfer of CD4 + CD25hi CD127− or CD4 + CD25 + FoxP3+ Tregs^2,3^. While promising, one concern about these efforts is that the transferred or activated Tregs are not specific to islet cell antigens. Non-specific tolerance induction may have off-target effects or may be too weak to rescue and preserve pancreatic islet cells. Targeting regulatory T cell activity to the islet antigens may improve both the efficacy and specificity of Treg treatment for T1D.

Tregs regulate immune responses to disease-specific antigens in the peripheral circulation^4^. Both adaptive and natural (nTregs) are activated by antigen-specific epitopes binding to the T cell receptor (TCR), down-modulating the T effector cell response to that same antigen. IL-10, TGF-β, and other cytokines are believed to be involved. Alternatively, direct cell-to-cell interactions between tolerogenic antigen presenting cells and effector T cells may contribute to antigen-specific tolerance induction^5^.

The exact peptides that induce Tregs to respond have been hard to define, and most of the Treg epitopes uncovered to date have been tested in T1D. For example, mycobacterial Hsp70-derived peptide B29 has been shown to activate regulatory T cells in clinical studies of T1D^6^ and arthritis^7^. In addition, two insulin-derived Treg-activating peptides have been identified: Insulin B9-23 (SHLVEALYLVCGERG) in NOD mice^8^ and C19-A3 (GSLQPLALEGSLQKRGIV) in NODs and humans^9^. C19-A3 therapy is progressing to the clinic in Phase I studies^10^, and initial reports appear to demonstrate that islet cell function is preserved in patients treated with the peptide, as compared to the no treatment group^9^. In contrast, development of B9-23 became more complicated when prolonged administration of the peptide induced anaphylaxis in NOD mice^11^; subsequent studies of this peptide suggest that the peptide-MHC binding is somewhat unstable and that the effect of the peptide depends on extrinsic factors^12^.

**Figure 1:**
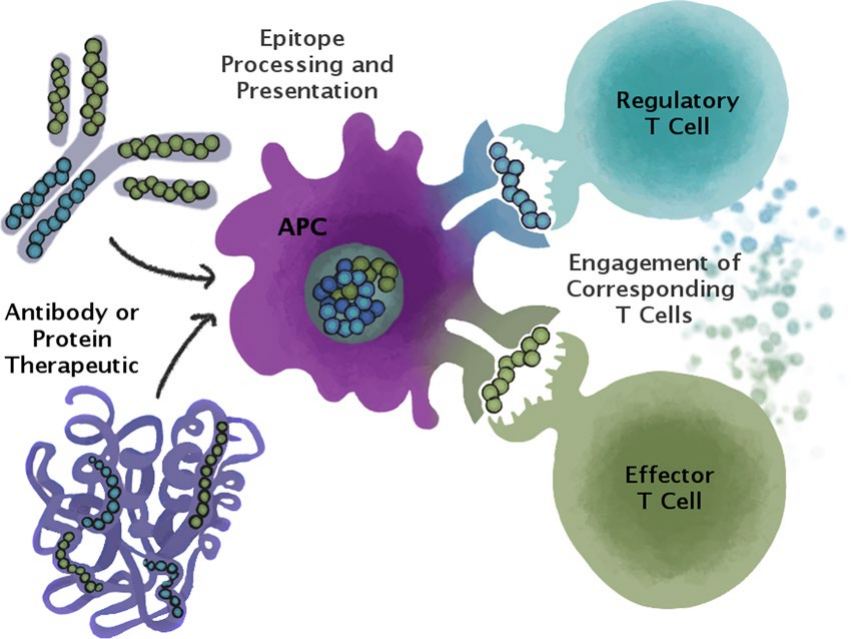
Antigen-Specific Adaptive Tolerance Induction (ASATI). Tregitopes are highly conserved regulatory T cell epitopes found in human and other species’ IgG (blue peptides in this illustration). They may serve to reduce the immunogenicity of neo-epitopes in the hypervariable region of the IgG CDR. We show that when Tregitopes are co-administered with other antigens (such as Type 1 Diabetes target antigens), they can suppress immune response to those antigens. Thus, Tregitopes have therapeutic potential in autoimmunity, allergy and in other clinical applications.

Antigen-specific adaptive tolerance induction (ASATI) may be boosted when antigens are administered in combination with regulatory T cell epitopes, such as Tregitopes (**T reg**ulatory cell ep**itopes**). Tregitopes are natural T cell epitopes derived from immunoglobulin G (IgG; Fig. 1 ^13^), which may also explain the regulatory effects of intravenous immunoglobulin (IVIG) therapy^14^. Tregitopes have the following properties: they (a) bind to multiple MHC class II molecules, (b) suppress effector T cell responses to co-delivered antigen, and (c) up-regulate Treg-associated cytokines and chemokines^13,15^.

The putative mechanism of action of Tregitopes has been described in previous publications^16,17^. Briefly, Tregitopes are processed and presented by antigen-presenting cells in close proximity to target antigens, activating Tregitope-specific Tregs; nearby target-antigen-specific T effectors are converted to induced regulatory T cells (iTreg) which are antigen-specific (Fig. 1).

Specific to the “ASATI” hypothesis under investigation here, previous studies in murine models have demonstrated the ability of Tregitopes to induce antigen-specific tolerance (including conversion of epitope-specific Teff to Treg) in DO11.10 mice^18^, to modulate antigen-specific transplant rejection^19^ and to reduce immune responses to allergens *in vitro* and *in vivo*^19,20^. T cells responding to Tregitopes exhibit a T regulatory phenotype in mice (CD4 + CD25 + FoxP3+)^13^.

We previously demonstrated that Tregitope peptides co-administered with preproinsulin (PPI) peptides (T1D-ASATI) could prevent and treat diabetes in NOD mice^18^. Tregitopes administered to NOD mice prior to the development of diabetes completely abrogated the development of diabetes compared to control groups (p < 0.01). Furthermore, when co-administered with the “target” antigen (five preproinsulin or PPI peptides) in liposomes, treatment with Tregitope peptides completely suppressed the development of T1D in NOD mice^18^. While the administration of Tregitope peptides (in IFA) in an additional therapeutic study was effective and an important proof-of-concept, this delivery vehicle is not suitable for translation to the clinic for T1D. The challenge of antigen-specific Tregitope therapy of T1D entails the development of an appropriate therapeutic delivery system for human use as well as optimization of both disease-specific antigen and Tregitope components.

The studies described here were designed to explore the hypothesis that Tregitopes, administered with target antigen (PPI peptides, T1D ASATI) would be more effective than Tregitopes alone, due to the direct action of Tregitopes on antigen-specific T cells and their conversion to antigen-specific Tregs (as was observed in DO11.10 mice in the previous publication^18^). Tregitope peptides were administered to NOD mice at the onset of diabetes within two potential human delivery systems (liposomes and in HSA-fusion products) in combination with PPI target antigen peptides. After demonstrating that HSA-Tregitope fusions were non-toxic, as compared to the well-established toxicity of HSA in NOD mice, we show that the combination of Tregitope-HSA fusions and PPI peptides reduce the incidence of severe diabetes and reverses mild diabetes, over the course of 49 days of treatment and observation.

## Materials and Methods

To model the application of Tregitope therapy in the clinical setting, where it is more likely that T1D-ASATI would be initiated after T1D is diagnosed, we co-administered Tregitope peptides with the target antigen following the establishment of diabetes in the NOD mouse model. We also tested a formulation of Tregitopes that had potential for extending the half-life of the peptides, a Tregitope-HSA fusion. All of the Tregitope peptides used in this study were human Tregitopes and were previously shown to have sufficient cross-species conservation to elicit Treg response in NOD and other murine models^15,18,19,21,22^.

### Tregitopes

Tregitopes are 15 to 20 amino acid long peptides derived from immunoglobulin^15^. Tregitope sequences and class II-binding control peptide sequences (“scrambled” Tregitopes) are listed in Table 1.

**Table 1:** T1D-ASATI Peptide Selection.

	Peptide ID	Sequence
Tregitope peptides	HTREG-IGGH-167	PAVLQSSGLYSLSSVVTVPSSSLGTQ
	HTREG-IGGC-289	EEQYNSTYRVVSVLTVLHQDW
	HTREG-IGGH-009A	GGLVQPGGSLRLSCAASGFTF
	HTREG-IGGH-029B	MHWVRQAPGKGLEWV
	HTREG-IGGK-084	GTDFTLTISSLQPED
	HTREG-IGGK-0134	LNNFYPREAKVQWKVDNALQSGNS
PPI peptides	Insulin B6-B20	LCGSHLVEALYLVCG
	Insulin S22-B12	AAAFVNQHLCGSHLV
	Insulin C34-A13	KRGIVEQCCTSICSL
	Insulin S1-S20	MALWMRLLPLLALLALWGPDP
	Insulin B22-C12	RGFFYTPKTRREAEDLQV
	Insulin C23-A2	LQPLALEGSLQKRGI
Scrambled Tregitope peptides	HTREG-IGGH-167-SCRAM	SVQGTSYVGSSLSAPPTSSLSVQLVL
	HTREG-IGGC-289_SCRAM	TSLWDSVTVVNEVYQRYHEQL
	HTREG-IGGH-009A_SCRAM	VLSPLGRAGCAGTLGSFSQGF
	HTREG-IGGH-029B_SCRAM	HAPKVQLMGVWEWRG
	HTREG-IGGK-084_SCRAM	TLISSTFEGQDDLTP
	HTREG-IGGK-0134_SCRAM	SKLSVLAFDVGPQNNQENRANWYK

Tregitope sequences that were used as peptides or incorporated into the HSA-fusions described in this study are listed in this table. Also listed are the preproinsulin (PPI) peptides and the sequences of class II-binding “scrambled” Tregitopes that were used as control peptides.

### PPI epitopes and clusters

The complete sequence of human PPI was analyzed by the EpiMatrix system and pre-pro-insulin (PPI) T cell epitopes were identified as described in reference^18^. PPI epitopes used in these studies are listed in Table 1. “H” denotes human Tregitopes; PPI peptides were also derived from the human sequence of insulin.

### Peptides and peptide synthesis

All peptides were synthesized by standard solid-phase 9-fluoronylmethoxy-carbonyl (Fmoc) synthesis by 21^st^ Century Biochemicals (Marlborough, MA, USA). Peptide purity of >85% was confirmed by analytical reversed phase HPLC and peptide mass determined by collision-induced dissociation and tandem mass spectrometry.

### Liposome preparation

Liposomes used in this study were manufactured by Juvaris BioTherapeutics, Inc (Pleasanton, CA). The liposomes were prepared by combining synthetically prepared cationic lipid, (1-[2-[9-(Z)-octadecenoyloxy]]-2-[8](Z)-heptadecenyl]-3-[hydroxyethyl]-imidazolinium chloride, termed DOTIM), and the neutral lipid cholesterol. The lipids were combined in equimolar concentrations (20 mM DOTIM/20 mM cholesterol) in tert-butanol, lyophilized, and then rehydrated in sterile water for injection (SWFI) to form liposomes. Liposomes were terminally filtered through a 0.2μm absolute filter into sterile containers and stored at 4 °C.

To encapsulate the Tregitope, Scrambled Tregitope and/or PPI peptides in the liposomes, lyophilized peptides, in DMSO, were added to the liposomes with gentle mixing via pipette. Sterile phosphate-buffered saline (PBS, Sigma) solution was added to liposome/peptide for final dosing of 25ug per peptide per treatment. The final DMSO concentration was less than 1%. DMSO was also included in the control liposomes, at the same final concentration, to control for potential DMSO effects in these *in vivo* studies.

### HSA fusion preparation

Under a collaborative agreement between EpiVax (Providence, RI) and Novozymes (Nottingham, United Kingdom), Tregitope sequences were fused to the C-terminal end of recombinantly-produced human serum albumin (Supplemental Fig. S2). The resulting DNA constructs were transformed into proprietary Saccharomyces cerevisiae yeast expression system (Novozymes, Nottingham). Several HSA-Tregitope fusions were produced (A-E). Tregitope-Albumin Fusion E, which contained Tregitopes 84 and 167, was produced in a sufficient quantity and at high enough quality (absence of truncations) for further use in mouse studies. Novozymes also provided similarly-produced recombinant HSA (no fusions) for the control arm of this study.

### *In Vivo* study methods

Two studies were performed: T1D ASATI therapy using Tregitopes in liposomes and T1D ASATI therapy using HSA-Tregitope fusions (Supplemental Fig. S1A–C). Prior to initiating the HSA-Tregitope fusion study, a pilot study was performed to identify the optimal dosing regimen for recombinant albumin in these highly HSA-sensitive mice (Supplemental Fig. S1B). The fully human HSA (control arm) proved to be toxic for NOD mice, whereas HSA-Tregitope fusions were not (both versions of HSA were produced in yeast). So as to preserve mice in the control arms of the HSA-Tregitope study, all mice enrolled into the therapeutic treatment (HSA-Tregitope fusion) or control (HSA) arms received split dosing as shown in the study protocol, Supplemental Fig. S1C.

#### Mouse models

Non-obese diabetic (NOD/ShiLtJ) mice, a polygenic model for T1D, were purchased from the Jackson Laboratory (Bar Harbor, ME, USA). NOD/ShiLtJ female mice exhibit diabetes beginning at around 16 weeks of age, characterized by insulitis, a leukocytic infiltrate of the pancreatic islets^23^. Plasma glucose levels were used to determine disease onset. All animals for both studies were housed and bred in pathogen-free micro-isolator cages at the animal facilities operated by The Jackson Laboratory West (Sacramento, CA.). This study was conducted by *In Vivo* Services at The Jackson Laboratory Sacramento facility, an OLAW-assured and AAALAC-accredited organization. This study was performed according to the Jackson Laboratory IACUC-approved protocol and in compliance with the *Guide for the Care and Use of Laboratory Animals* (National Research Council, 1996 [Liposome study] and 2011 [HSA study]).

#### Tregitope and insulin peptide liposome delivery study

One-hundred and thirteen NOD/ShiLtJ (JAX# 1976) female mice were monitored for onset of diabetes by weekly urine testing for glycosuria from 8 weeks of age. Initially, when two consecutive positive glycosuria results occurred, caretakers immediately performed a BG measurement to identify mice that had BG levels between 200–300 mg/dl for study entry. The majority of NOD mice identified with these criteria had progressed beyond the 300 mg/dl at the time of randomization. Therefore, the study protocol was amended to perform two BG measurements weekly to confirm diabetes onset (and that blood glucose levels remained between 200–350 mg/dl prior to study entry). Treatments were initiated following confirmation of diabetes onset and mice were enrolled into one of six study groups (G1–G6) on a rolling basis.

Each study group received a total of six treatments, one on each of the following study days: 0, 7, 14, 35, 42 and 49, via subcutaneous injection with combinations of PBS (vehicle control with identical DMSO concentration as the peptides), PPI peptides and/or Tregitopes or scrambled Tregitope peptides (control). The PPI peptides (in 1%DMSO/PBS) were emulsified and delivered in liposomes with/without Tregitopes (See Supplemental Fig. S1A). Cage-side observations were made on a daily basis, and non-fasting blood glucose, clinical status and weights were assessed twice weekly. Mice were euthanized if their BG stayed above 600 consistently (BG ≥ 600 5 times), if they lost excessive body weight (>20% of initial body weight), or if they were found to be in a moribund state by the caretaker.

#### Human serum albumin toxicity pilot study

Human serum albumin (HSA) administration to NOD mice has been observed to result in a high frequency of anaphylaxis and death^24^. So as to preserve mice in the control arm, we evaluated the optimal dosing regimen for HSA in a pilot study, with the aim of limiting the potential for anaphylaxis in the control (HSA-treated) mice. Six NOD/ShiLt/J female mice (JAX# 1976) were enrolled into each of four groups: Group 1, HSA administered subcutaneously (SC) alone as a single dose (800ug/100uL on days 0, 13, 27 and 41); Group 2, HSA delivered SC as a split dose (400ug/100uL) one day apart (day 0, 1, 13, 14, 27, 28, 41 and 42); Group 3, HSA delivered SC as a split dose in left and right flanks (400ug/100uL to each flank on days 0, 13, 27 and 41) and Group 4, HSA delivered SC as a single dose (800ug on days 0, 13, 27 and 41) following pretreatment with an antihistamine, diphenhydramine (25 mg/kg given 15 minutes prior to HSA, administered subcutaneously, on days 13, 27 and 41). For injections that occurred after Day 0, cage side observations were carried out at 1, 2, and 4 hours post injection so as to identify any signs of drug toxicity in mice. Mice were then observed daily, over the following 49 days post-treatment.

#### HSA-Tregitope fusion treatment study

For this study, we modified the recruitment protocol so as to limit the number of NOD mice that could not be enrolled due to rapid progression of diabetes (see above). Beginning at 12 weeks of age, 246 NOD/ShiLtJ (JAX# 1976) female mice had BG monitoring twice weekly using a hand-held glucometer. Mice that had BG values between 200–350 mg/dl were retested as early as possible on the following day and those with a second confirmed BG level between 200–350 mg/dl were enrolled into the study immediately.

Mice were assigned to seven study groups on a rolling basis until each group contained 12 mice. Mice in groups 2–6 received split dosing of the treatments (400ug/100ul/mouse) on days 0/1, 13/14, 27/28, and 41/42 via subcutaneous injection; mice in group 1 (G1) received no treatment and mice in group 7 (G7) received single dose treatment (800ug/100ul/mouse) on days 0, 13, 27 and 41. Each mouse remained in the study for 49 days after the initial dose of treatment. Clinical observations and body weight measurements were performed on a weekly basis. For injections that occurred after study Day 0, cage side observations were carried out at 1, 2 and 4 hours post injection to identify any signs of toxicity due to the treatments. Mice that appeared moribund, as demonstrated by hunched posture, unkempt fur or weight loss exceeding 20%, were euthanized.

### Statistical analysis

BG measurements across all available mice were compiled for a time-series analysis. Mice which reached a study endpoint that was not defined by BG (moribund) were assigned the maximum BG level (600). Differences between groups at each timepoint and differences in the temporal change in BG measurements were evaluated using (i) the t-test, (ii) Mann-Whitney U test and (iii) the nonparametric difference test using 1,000 permutations. Comparisons of the absolute BG levels between two groups at each time point were considered statistically significant at a p-value < 0.05.

#### Q-TWiST

In this analysis that is intended to reflect quality-of-life, a series of disease states corresponding to clinically relevant conditions were defined and paired with weights known as utility coefficients. These weights have values between 0 and 1 and correspond to the perceived quality-of-life of each state, ranging from death to perfect health, respectively. Q-TWiST measures were calculated by summing the adjusted lengths of time spent in each state, weighted based on their respective utility coefficient. A general form of the Q-TWiST measure can be defined as:

$${\rm{Q}} \mbox{-} {\rm{TWiST}}=\sum \,_{i}{u}_{i}{T}_{i},$$

where *u*_*i*_ and *T*_*i*_ correspond to the utility coefficient of state *i* and to the time spent in state *i*, respectively. Assigning every utility coefficient to 1 is equivalent to following overall survival times without considering quality-of-life data.

Q-TWiST measures were first generated for each mouse according to the disease states and utility coefficients defined in Table 2. Survival times were partitioned into three states, healthy, diabetic, and severely diabetic, based on mouse BG levels. The total amount of time spent in each disease state was measured for each mouse enrolled in the study. Raw survival times were first averaged for each treatment group and then weighted using the defined utility coefficients.

**Table 2:** Q-Twist Analysis Parameters.

Disease State Tier	Description	Definition	Utility Coefficient
1	Healthy	BG ≤ 200	1
2	Diabetic	200 < BG ≤ 500	0.6
3	Severely diabetic	BG > 500	0.2

Quality of life parameters used in the Q-TWiST analysis.

BG = blood glucose, measured as mg/dL.

Please see supplemental data and figures for this study in the manuscript supplement. Additional details for the methods, including the raw data and details on the analysis for this study are available from the corresponding author.

## Results

### Tregitopes + PPI peptides in liposomes (Study 1)

**Figure 2:**
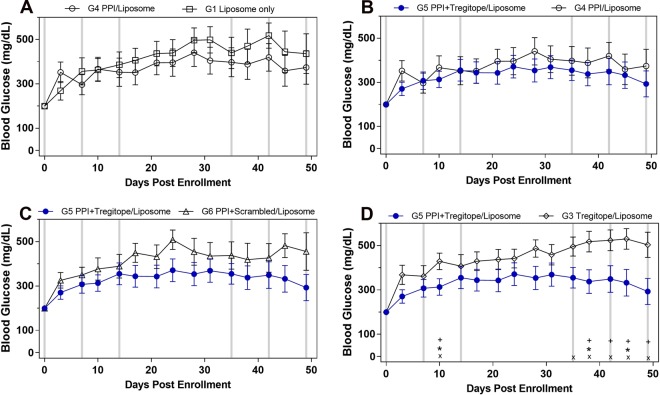
Blood glucose time-course comparisons, liposome delivery study. Mice were enrolled onto this study at a blood glucose range between 200–350 mg/dL. Light grey vertical bars represent treatment points at enrollment Day 0, Day 7, Day 14, Day 35, Day 42 and Day 49. Blue lines with solid circles represent the treatment group (PPI peptides + Tregitopes, G5) and black lines with open symbols represent control groups (PPI only, Liposomes only, PPI peptides with scrambled Tregitopes, or Tregitopes only). All peptide test articles were formulated in liposomes. Statistically significant differences (P < 0.05) in the absolute BG levels between groups are represented at the bottom of each graph. ^+^t-test; *Mann-Whitney; ^x^nonparametric-difference.

Sixty-five mice were enrolled into the treatment phase after a confirmed non-fasting BG of 200–350 mg/dl. By day 49, 54–80% of the mice in each of the arms reached an endpoint of a BG of 600 mg/dl or greater, or death (the expected rate of severe diabetes at this time point is 80%). Mean BG over time showed similar trends in the following control groups in Fig. 2: Group 1 (G1) liposome alone (containing vehicle, Fig. 2A open squares), G4 PPI alone (Fig. 2A,B, open circles), G6 PPI with scrambled Tregitope (Fig. 2C, open triangles) and G3 Tregitope alone (Fig. 2D, open diamonds) as compared to T1D-ASATI (G5). *In vivo* study designs are shown in Supplementary Fig. S1A–C.

In contrast, the group of mice receiving the T1D-ASATI treatment, consisting of Tregitope with PPI peptides (Fig. 2D, G5, closed circles) had significantly lower mean BG than Tregitope (no PPI) alone (Fig. 2D, G3) open diamonds), particularly from days 35 to 49. As shown in Fig. 2C, the Tregitope with PPI peptide T1D-ASATI group (G5) also had consistently lower mean BG levels than the control (G2, PPI + Scrambled Tregitope) group, however, those differences did not reach statistical significance. In comparisons of the Tregitope with PPI T1D-ASATI group against the individual controls (G2, PPI + Scrambled Tregitope, Fig. 2C and G3, Tregitope no PPI, Fig. 2D), the BG over time curves are seen to diverge early in treatment and to progressively outperform the control group through day 49.

**Figure 3:**
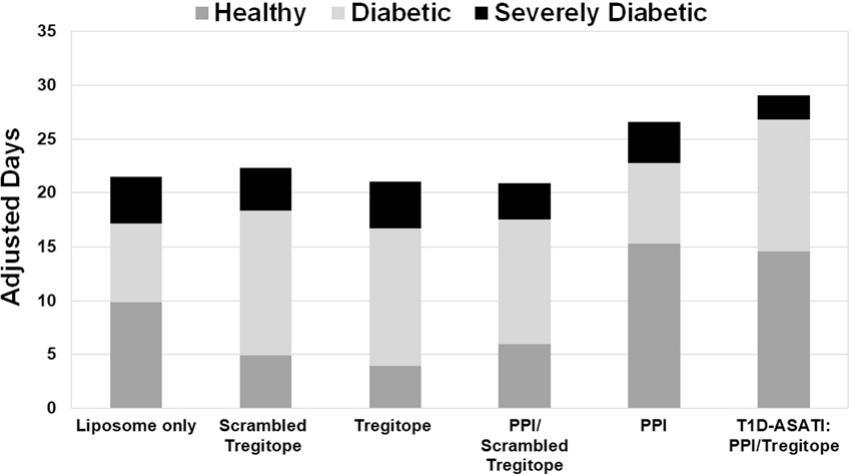
Quality of Life Adjusted Survival Time (Q-TWiST) for Liposome Tregitope-PPI NOD Study. The total amount of time in each disease stage (as defined by BG level) was measured for each mouse enrolled in the study. Three states were characterized as healthy (dark grey, BG ≤ 200); diabetic (light grey, 200 < BG ≤ 500); severely diabetic (black, BG > 500).

The Quality of Life Adjusted Survival Time (Q-TWiST) analysis illustrates the overall longest survival and a longer duration of time in a favorable clinical state in the Tregitope with PPI peptides (T1D-ASATI) group, characterized by lower BG levels even after having started at BG > 200. This analysis also highlights the key contribution of Tregitopes in the Tregitope with PPI peptides T1D-ASATI experimental treatment arm, as there was no effect of PPI when administered with the scrambled peptide controls for the Tregitope peptides. Figure 3 demonstrates the potential impact of T1D-ASATI combination therapy in the clinical setting: Mice in the liposomes with Tregitope with PPI peptides group (T1D-ASATI, G5) spent the greatest amount of time in a more favorable clinical state (BG ≤ 500) and the least amount of time in a state of severe diabetes (BG > 500). See Supplementary Fig. S3 for a heatmap of individual mouse blood glucose data for each measurement over the entire liposome delivery study.

### HSA-Tregitope fusions + PPI peptides (Study 2)

#### HSA-Tregitope fusions

**Figure 4:**
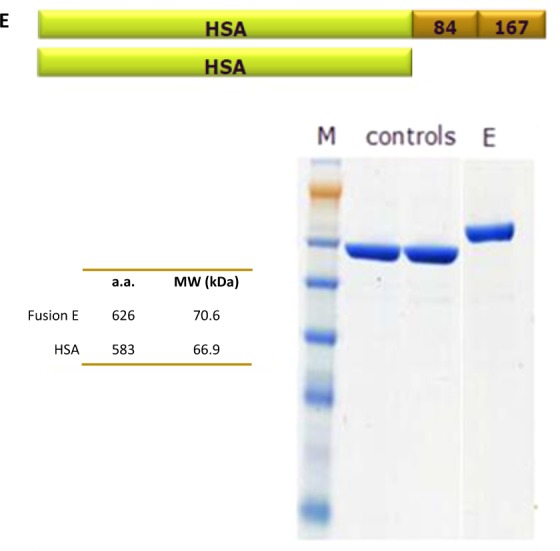
Structure and characterization of HSA-Fusion E (Tregitope) protein. The HSA and the HSA-Fusion E (Tregitope) protein, were produced in yeast by Novozymes. The design of the HSA-fusion construct, its molecular weight (70.6) compared to HSA (66.9), and the final products (gel electrophoresis) are shown.

Several different versions of HSA-Tregitope fusions were designed and produced in collaboration with Novozymes (Nottingham, UK). HSA-Tregitope Fusion E contains human Tregitopes 167 and 84, was very well expressed and produced in sufficient quantity. Fusion E was selected for evaluation in the second NOD study (Fig. 4, see also Supplemental Fig. S2).

#### Selection of control dosing schedule for human albumin (HSA)

**Figure 5:**
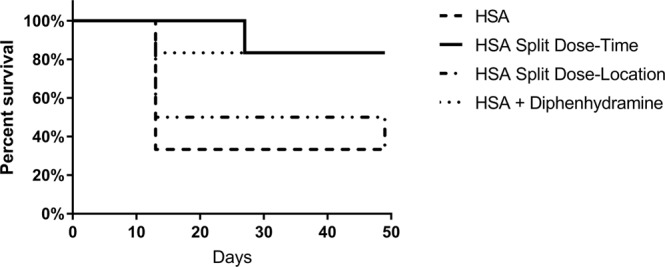
HSA Toxicity Study in NOD Mice. Six NOD mice were enrolled in each of four study groups. Group 1 received HSA as a single dose (dashed line) at the same intervals used in the HSA-Tregitope Fusion study. Group 2 received HSA as a split dose, one day apart, Group 3 received HSA delivered SC as a split dose in left and right flanks on the same day, and Group 4 received HSA as a single dose following pretreatment with an antihistamine, all at the same intervals as were used in the final HSA-Tregitope fusion study. Single dose HSA resulted in the highest mortality rate after the second dose (67%, 4/6 mice); split dose (by day) resulted in the lowest morbidity and mortality rate (1/6 mice after the third dose) and this regimen was therefore selected for the study.

In unpublished studies in our own laboratory and in published studies, it has been demonstrated that the repeated administration of certain human proteins such as Alpha-1-Antitrypsin and Albumin to NOD mice may result in anaphylaxis^24^. To mitigate this potential complication in the planned study, in which we would be administering novel HSA-peptide fusions, we performed a pilot toxicity study using HSA alone (which would serve as the control for the HSA-peptide fusion, Fig. 5**)**.

The study design is shown in Supplementary Fig. S1B. Mice were administered HSA a single dose subcutaneously, as a split dose one day apart also subcutaneously, as a split dose in left and right flanks on the same day, or pretreated with an antihistamine, diphenhydramine. All treatments were repeated weekly. Deaths were recorded, and these occurred only on the second or later dose of therapy. Mice who died developed illness (lethargy, prostration) within 1–2 hours of dosing. 67% of mice in the HSA single dose control arm (G1) died soon after the second dose of HSA. Antihistamine pre-treatment (G4) reduced mortality (from 67% to 17%), however, all mice developed antihistamine injection site reactions, characterized by hair loss and skin irritation. Split HSA dose by location (administered on the same day, G3) did not reduce mortality (67%). Split dose by time (two doses of HSA, one day apart, G2) led to the most favorable outcome, with good tolerance and reduced mortality. Only one HSA-associated death was observed, at the third time point (17%). Based on these results, the split dose by time was chosen for both HSA (control treatment) and HSA-Tregitope fusion in the planned HSA-Tregitope treatment study.

#### *HSA-Tregitope fusion treatment*

**Figure 6:**
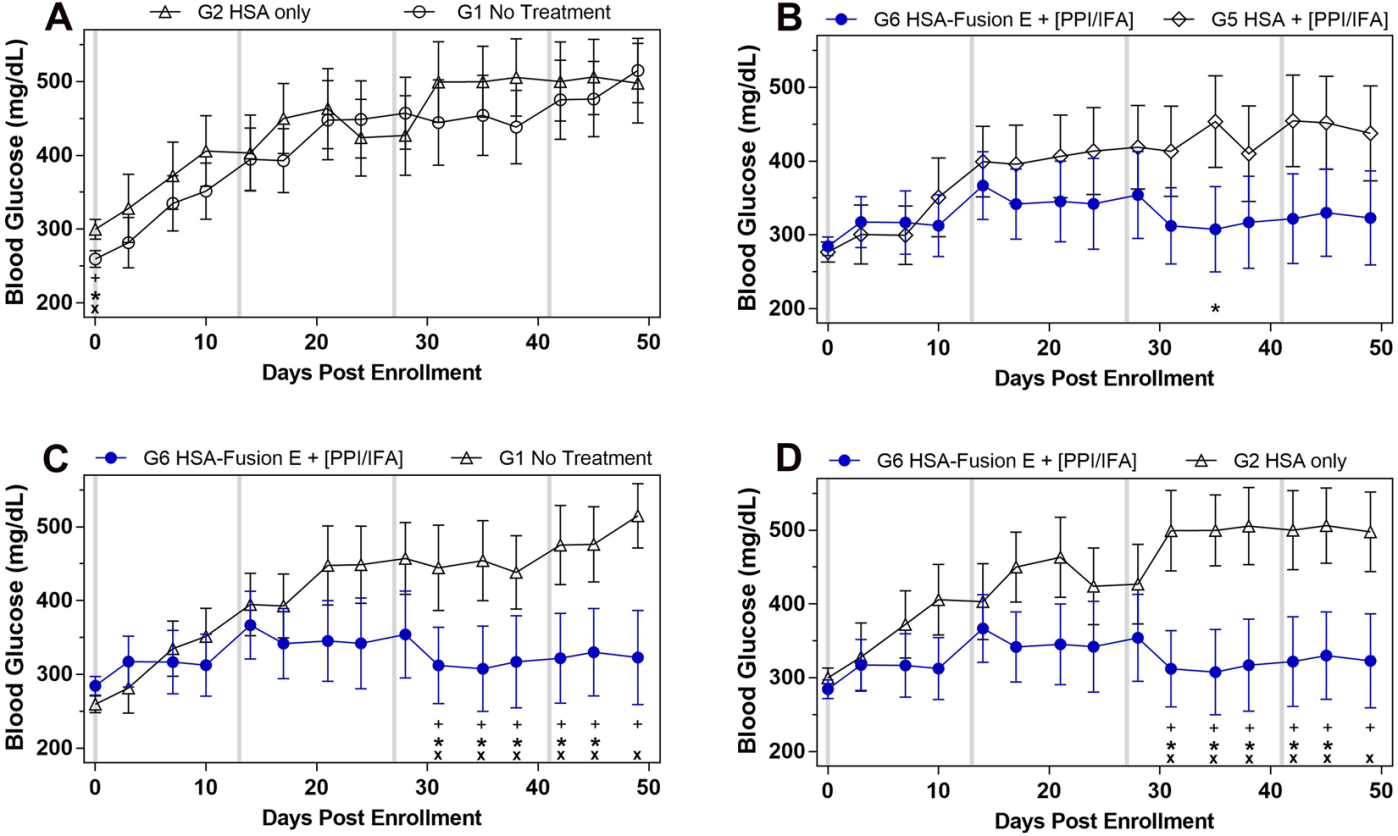
Mean blood glucose time-course comparisons for HSA-Tregitope fusion study. Beginning at 16 weeks of age NOD/ShiLtJ mice were tested for BG levels two times per week. When BG was registered between 200–350 mg/dL on two consecutive days, mice were assigned to one of seven study groups on a rolling basis until each group contained 12 mice. Vertical bars represent treatment points. All groups shown here received split dose treatments of HSA or HSA-Tregitope fusions, split between two consecutive days. Blue lines with solid circles represent the treatment group (G6, HSA-Fusion E + PPI peptides) and black lines with open symbols represent comparison control groups (G1, No Treatment, HSA only, HSA + PPI). Statistical differences between the absolute BG levels between the groups were determined as p-value < 0.05, is represented in the bottom of each graph. ^+^t-test; *Mann-Whitney; ^x^nonparametric-difference.

A total of 84 mice were enrolled into the treatment phase after a confirmed non-fasting BG of 200–350 mg/dl. The study design is shown in Supplementary Fig. S1C. By day 49, 33–75% of the mice (by treatment group) reached an endpoint of a BG of 600 mg/dl or greater, or death (Fig. 6).

HSA-Tregitope Fusion E was very well tolerated by NOD mice in this study, even when administered as a single dose (0% mortality after the second dose of HSA-Tregitope in the single dose arm G7). This is dramatically different from single dose HSA in the toxicity study (67% mortality). This is consistent with our hypothesis that Tregitopes may actively suppress effector T cell responses^17^ and confirms our observation in another study that anaphylactic responses to HSA were reduced when Tregitopes were fused with HSA (unpublished, EpiVax). Split-dose administration of HSA in the control group also completely prevented HSA-induced anaphylaxis in these NOD mice.

#### HSA (Control) treatment impact on diabetes. 

The No Treatment (G1) and (G2) HSA-alone (Vehicle control) groups showed similar trends in mean BG over time (Fig. 6A), with 75% of each group reaching an endpoint of BG ≥600 or death. PPI peptides appeared to have some effect on the development of diabetes, as mice treated with HSA plus PPI peptides (G5, open diamonds, Fig. 6B) also had slightly improved BG trends compared to controls in Fig. 6A, however this effect was significantly improved by the addition of Tregitopes in the HSA-fusion E + PPI group (G6, closed circles, Fig. 6B).

#### T1D-ASATI treatment impact on diabetes

Mice belonging to the HSA-Fusion E + PPI group (T1D-ASATI, G6, closed circles) were more often able to control their BG over time, with a statistically significant difference in comparison to G1, the No Treatment arm by day 49 (Fig. 6C). The mean BG was also lower in the G6 HSA-Fusion E + PPI (T1D-ASATI) group in comparison to the G2 HSA alone group, with statistically significant differences from day 31 to day 49 (Fig. 6D). As there appeared to be some exacerbation of diabetes in the HSA alone group, these differences between the HSA-Fusion E + PPI were more marked in comparison to the HSA alone group than to the No Treatment group.

Analysis of individual BG measures for each mouse revealed some notable findings. First, while progression of diabetes was seen in all groups, the onset of severe diabetes (BG > 600) or death occurred at a lower frequency and with delayed onset in the treatment groups that received Tregitopes, PPI peptides, or both. Accelerated development of diabetes was observed in the mice that received HSA (G2, Fig. 6A). Second, some spontaneous control was observed in all groups, including the No Treatment group (G1). Greater numbers of animals were able to control their diabetes (BG <200) by day 49 in the active treatment groups (Tregitope, G4, PPI peptides, G5 or both, G6) compared to the No Treatment (G1) or HSA-alone control groups (G2). See Supplementary Fig. S4 for a heatmap of individual mouse blood glucose data for each measurement over the entire HSA-Tregitope fusion study.

**Figure 7:**
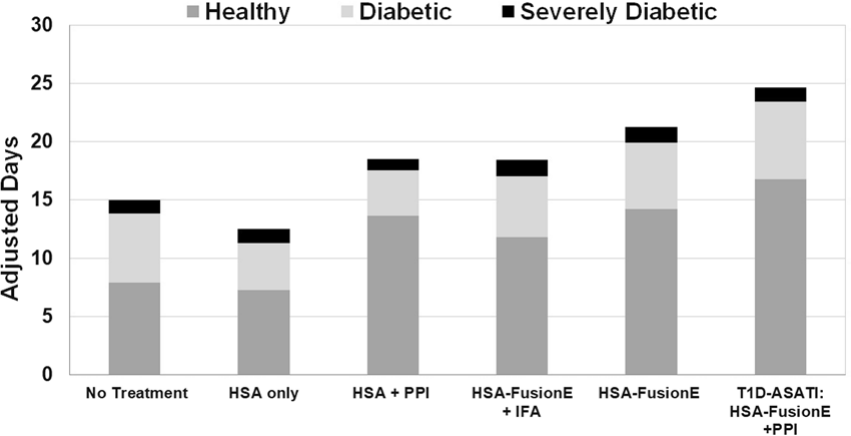
Quality of Life Adjusted Survival Time (Q-TWiST) for HSA-Fusion E NOD Study. The total amount of time in each disease stage (as defined by BG level) was measured for each mouse enrolled in the study. Three states were characterized as healthy (dark grey, BG ≤ 200); diabetic (light grey, 200 < BG ≤ 500); severely diabetic (black, BG > 500).

The relative proportions of time spent in favorable versus unfavorable clinical states for the study using HSA-Tregitope fusions were further illustrated in the Quality of Life Adjusted Survival Time (Q-TWiST) analysis (Fig. 7) using the definitions from Table 2. This graphical representation of quality-of-life demonstrates the HSA-Fusion E + PPI group remained in favorable categories for the longest duration of time as compared to the other treatment groups. Survival was also characterized by the longest duration of BG levels in the normal range (<200, healthy) as compared to other groups.

Table 3 illustrates the frequency of unfavorable and favorable outcomes by group. As is illustrated in the BG heatmaps and summarized here, the HSA alone control group had the highest proportion of mice reaching either of two unfavorable endpoints: death (75%) and death or a BG of 600 mg/dl or greater (75%). In contrast, these adverse outcomes were seen in only 33% of the HSA-Fusion E + PPI group.

**Table 3:** Outcomes as Proportion of Study Group for HSA-Fusion E NOD Study.

FAVORABLE and UNFAVORABLE OUTCOMES by GROUP	No Treatment	HSA only	HSA + PPI	HSA-FusionE + IFA	HSA-FusionE only	T1D-ASATI: HSA-FusionE + PPI
Proportion reaching endpoint - death	58%	75%	58%	50%	33%	33%
Proportion reaching endpoint -death or BG ≥ 600	75%	75%	67%	50%	50%	33%
Proportion alive with BG < 200 at the end of study	8%	8%	33%	33%	33%	50%
Proportion with at least 100 mg/dL drop in BG from peak levels	17%	25%	25%	33%	17%	67%

For each treatment group, outcome was determined by the number of mice in that outcome state of all mice in that treatment group. Blood Glucose (BG) over 600 was the pre-selected endpoint for this study. An equal number of mice (12) were enrolled into each group and the proportion of mice surviving is provided. HSA-Tregitope fusion G7 is not included in this table.

Two favorable outcomes are listed in Table 3. First, consider the proportion of mice in each group who were initially diabetic (at study entry) but ended the study with a normal BG (<200 mg/dl). Only one of 12 mice (8%) in the No Treatment and HSA alone groups ended the study with a normal BG, in contrast to six of 12 mice (50%) in the T1D-ASATI group. In addition, more mice in the T1D-ASATI arm were noted to regain control of their blood sugar after significant elevations. Using a threshold of a decline in BG of at least 100 mg/dl from peak values, this level of control was seen in eight of 12 mice (67%) in the HSA-Fusion E + PPI group. No more than four mice in any of the other treatment or control groups exhibited this level of BG control.

Supplemental Fig. S5A,B show the average BG for each of the arms in the study groups for each of the two studies. Supplemental Fig. S6 shows BG over time curves for individual mice who achieved a normal blood glucose (<200) by the end of the study (day 49). There was a much higher (6-fold) rate of achieving this resolution of diabetes in the HSA-Fusion E + PPI group vs. No Treatment or HSA only groups. A review of individual mouse weights demonstrated that lower BG levels were not related to diabetes-associated anorexia, as all of the mice resolving their diabetes gained weight or had minimal weight loss over the course of the study (No Treatment: −1.7%; HSA only: +7.4%, HSA-Fusion E + PPI/IFA: −1.7% to + 5.85%).

## Discussion

Type 1 diabetes is a lifelong disease, with onset predominantly in children and young adults. Data from large epidemiologic studies worldwide indicate that the incidence of T1D has been increasing by 2–5% and that the prevalence of T1D is approximately 1 in 300 in the United States by 18 years of age^25^. Risk for the development of T1D is affected by race, sex, geographic location and genetic factors. T1D also bears an association with other autoimmune disease, such as autoimmune thyroid disease, Addison’s disease, celiac disease, and autoimmune gastritis^25^, as the pathogenesis is mediated by autoreactive T cells. Currently approved treatments for T1D are limited to life-long insulin replacement therapy. Newer therapies, such as Glucagon-like peptide-1 (GLP-1) receptor agonists (also known as incretin mimetics) have been studied as an adjunct to insulin in T1D, with mixed results^26,27^. However, these newer therapies do not address the underlying autoimmune cause of ongoing beta islet cell destruction.

Given the limited future options of pharmacologic therapy, there is intense interest in addressing T1D as an autoimmune disease by specifically targeting the autoreactive T effector cells that mediate the destruction of beta cells in the pancreas. One insight into this type of therapy is the “honeymoon period” after T1D diagnosis, in which the pancreas is able to produce sufficient endogenous insulin to delay the need for insulin replacement. During this period, ongoing beta cell destruction may be an ideal target for early intervention with immune suppressive therapies. Broadly immunosuppressive therapies, such as antibody to CD3^28^ and anti-thymocyte globulin are currently in human clinical trials. However, collateral effects on immune responses to non-self-antigens may limit the long-term clinical applicability of these therapies.

A more specific target for immune intervention is the reconstitution of natural T regulatory (Treg) cell responses with the potential to dampen the autoreactive T effector cell-mediated beta cell destruction^29^. Development of a strong, beta cell-specific Treg response requires administration of Treg therapy with target antigens, such as peptides derived from or related to insulin. The insulin peptide, C19-A3, may be responsible for the honeymoon period that is frequently associated with the initiation of insulin therapy in T1D^30^ as it has been shown to induce Tregs in HLA-Transgenic (DR4) mice. This peptide is included in the set of peptides used in the PPI treatment arms in this study and may have contributed to improved outcomes observed when combined with Tregitope.

Other interventions for early diabetes include strongly agonistic insulin mimotopes, which have been used in humanized mice to tolerize and treat diabetes^31,32^. Administered conjugated to gold beads, and delivered by microneedle, this therapy is in Phase I studies. Other studies have targeted proinsulin, to enhance proliferation of Foxp3+, CD25^hi^ Tregs^33^. Even if Treg induction to target antigens such as insulin peptides in T1D patients is somewhat effective, it is quite clear that the impact of “tolerogenic therapy” with insulin itself is not long lasting (hence honeymoon, and not cure). Thus longer-lasting or more potent Treg induction is needed to bring enhanced success.

We have developed an interest in a Treg-activating set of peptides known as Tregitopes, first published in 2008^13^. Tregitope peptides have been shown to be effective in prophylactic and therapeutic studies conducted in T1D mice^18^, in transplant^17^ and in standard murine models in which tolerance has been evaluated^34^. In combination with disease-specific antigens, Tregitopes stimulate what we have termed antigen-specific adaptive tolerance induction, or ASATI. Specifically, in T1D, we have previously shown that Tregitopes, administered together with preproinsulin (PPI) peptides in liposomes, completely suppressed the development of T1D in pre-diabetic NOD mice^18^. Given that this formulation of Tregitopes is unsuitable for human studies, we are investigating alternative delivery vehicles for use in translation to human trials.

In this study, we first evaluated the combination of six Tregitope peptides in combination with six PPI peptides in liposomes in a NOD mouse model. PPI alone and Tregitope alone groups had similar mean blood glucose trends over time as the liposome control arm. The contribution of the antigen-specific (PPI) component of the therapeutic arm is demonstrated by significantly lower mean blood glucose measurements in the group treated with both PPI peptides and Tregitope peptides, compared to Tregitopes alone. The contribution of the Tregitope component of the combination is evident in lower glucose measurements in the PPI/Tregitope group as compared to a control arm of PPI peptides with scrambled Tregitope peptides. This overall more favorable course of diabetes is illustrated by the Q-TWiST analysis, with the PPI/Tregitope arm having the longest survival, characterized by the greatest proportion of time spent in the favorable clinical state with blood glucose levels less than or equal to 500 mg/dl.

We then proceeded to test Tregitopes expressed as a fusion protein with HSA in the same NOD mouse model. Several attempts at producing longer strings of Tregitopes fused to HSA were unsuccessful. However, two Tregitope peptides, 167 and 84, were successfully expressed as HSA-fusions and purified, and administered with six PPI peptides in the study reported here.

To establish a baseline for comparison, we performed a preliminary study of HSA administration in NODs. In previous studies using human proteins in NOD mice (as compared to other laboratory strains), there is a well-documented risk of anaphylaxis. We evaluated several methods of reducing the risk of anaphylaxis, reasoning that the development of immune-complexes might be the cause of the problem. We were able to safely administer HSA to NOD mice as a split dose, one day apart, with no incident of anaphylaxis in the course of the study. While we also performed split-dose treatment for the purpose of standardizing the control and study arms, we note that single-dose HSA when administered as an HSA-Tregitope fusion did not induce anaphylaxis in this study. We hypothesize that the presence of the immune modulating Tregitopes modified the potential for anaphylaxis from the co-administered HSA. This same effect of Tregitopes on HSA-related anaphylaxis was also observed in an unpublished study of HSA (control) vs. HSA-Tregitope fusions in separate studies (also using the NOD mouse strain).

In this split dose administration study, we also observed that HSA treatment exacerbated the progression of diabetes in NOD mice to some extent. We suspect this is consistent with a spectrum of inflammatory effects of HSA in NOD mice; while anaphylaxis was avoided using the split dosing regimen, the autoimmune destruction of pancreatic beta cells may have nonetheless been accelerated by the presence of an inflammatory reaction to the HSA in the lymph nodes of treated mice. This progression of diabetes was reduced when PPI peptides were added to HSA (peptide treatment), when HSA was administered as fusion protein with Tregitopes (Treg/Tolerance induction) and further reduced when both PPI peptides and the HSA-Tregitope fusions were used in combination (antigen-specific adaptive tolerance induction, ASATI).

These experiments do not provide direct evidence that protection against diabetes using PPI/Tregitopes is due to co-presentation by the Tregitopes with the target antigen on antigen presenting-cells (APC), as illustrated in Fig. 1. In the case of liposome co-delivery, presentation by the same APC is conceivable. However, co-delivery in the context of the second study (Tregitope-HSA) is more difficult to validate. Indirect evidence that co-presentation is required for efficacy is afforded by comparing HSA-Tregitope + PPI arms with control arms (HSA/PPI, or Tregitope/IFA).

Careful analysis of the time course of progression of diabetes in each group demonstrated a clear effect of PPI peptides plus HSA-Tregitope fusions. While a reduction in progression of diabetes was seen in the control groups of PPI peptides alone and HSA-Tregitope fusions alone, the greatest effect was seen in the HSA-Tregitope fusions plus PPI peptides, or T1D-ASATI group. The impact is evident in a comparison of blood glucose heat maps of individual mice, as well as in the comparison of mean blood glucose over time in each group, with the latter measurement demonstrating significantly lower blood glucose measurements, particularly after day 31. When evaluated in terms of unfavorable outcomes per group, the HSA-Tregitope fusion plus PPI group had the lowest proportion of mice reaching either the clinical endpoint (blood glucose of 600 mg/dl or greater) or death. Conversely, when evaluated in terms of favorable outcomes, the HSA-Tregitope fusion plus PPI group had the largest proportion of mice that were alive with a blood glucose less than 200 (healthy, non-diabetic) at day 49 or who exhibited at least a 100 mg/dl drop in blood glucose from a peak measurement. In addition to the visually striking difference in the blood glucose heat maps over time, this favorable outcome is also evident in the Quality-of-Life Adjusted Survival Time (Q-TWiST) analysis.

We further noted that a few mice in all groups had resolution of diabetes over the course of the 49-day study. The proportion of diabetes resolution, however, was markedly greater (6-fold) in the HSA-Tregitope fusion plus PPI group than in the No Treatment or HSA alone control groups. We noted these normal blood glucose levels were not accompanied by clinical signs of advancing diabetes (i.e., anorexia or weight loss). The HSA-Tregitope Fusion E plus PPI group also had the largest proportion of mice who demonstrated significant control of high blood glucose (at least a 100 mg/dl drop from peak) compared to all other groups. As shown in Supplemental Fig. S7, insulitis also trended lower for mice in groups that had improved control over blood glucose.

Taken together, these studies provide support for the therapeutic effect of T1D-ASATI, the combination of Tregitopes (as peptides in liposomes or as HSA-Tregitope-fusions) with diabetes specific antigen, PPI peptides. Multiple controls support our hypothesis of ASATI, since neither Tregitope peptides alone nor PPI peptides alone resulted in the same clinical outcomes. Directly linking T1D antigens to Tregitopes (by chemical linkage or molecular fusion) may improve the efficacy of Tregitope-PPI combination therapy.

In summary, Tregitopes appear to be more effective as fusion proteins (with albumin) in combination with target antigens than when used alone. This study suggests that T1D-ASATI may have promise for translation to the clinic.

## Supplementary information

Supplementary Information
