# Supplementary material for: Therapeutic administration of Tregitope-Human Albumin Fusion with Insulin Peptides to promote Antigen-Specific Adaptive Tolerance Induction
Source: Sci Rep. 2019 Nov 6;9:16103. doi: 10.1038/s41598-019-52331-1 (PMC6834854; doi:10.1038/s41598-019-52331-1)
Supplement: Supplementary file 1 — Supplementary Information [file 41598_2019_52331_MOESM1_ESM.pdf]

Therapeutic administration of Tregitope-Human Albumin Fusion with Insulin Peptides to promote Antigen-Specific Adaptive Tolerance Induction

Anne S. De Groot, Gail Skowron, James Robert White, Christine Boyle, Guilhem Richard, David Serreze, William D. Martin

Supplementary Information

Supplemental Figure S1. Study Designs for NOD mouse experiments

S1A. Tregitope and PPI (Insulin Peptide) Liposome Study

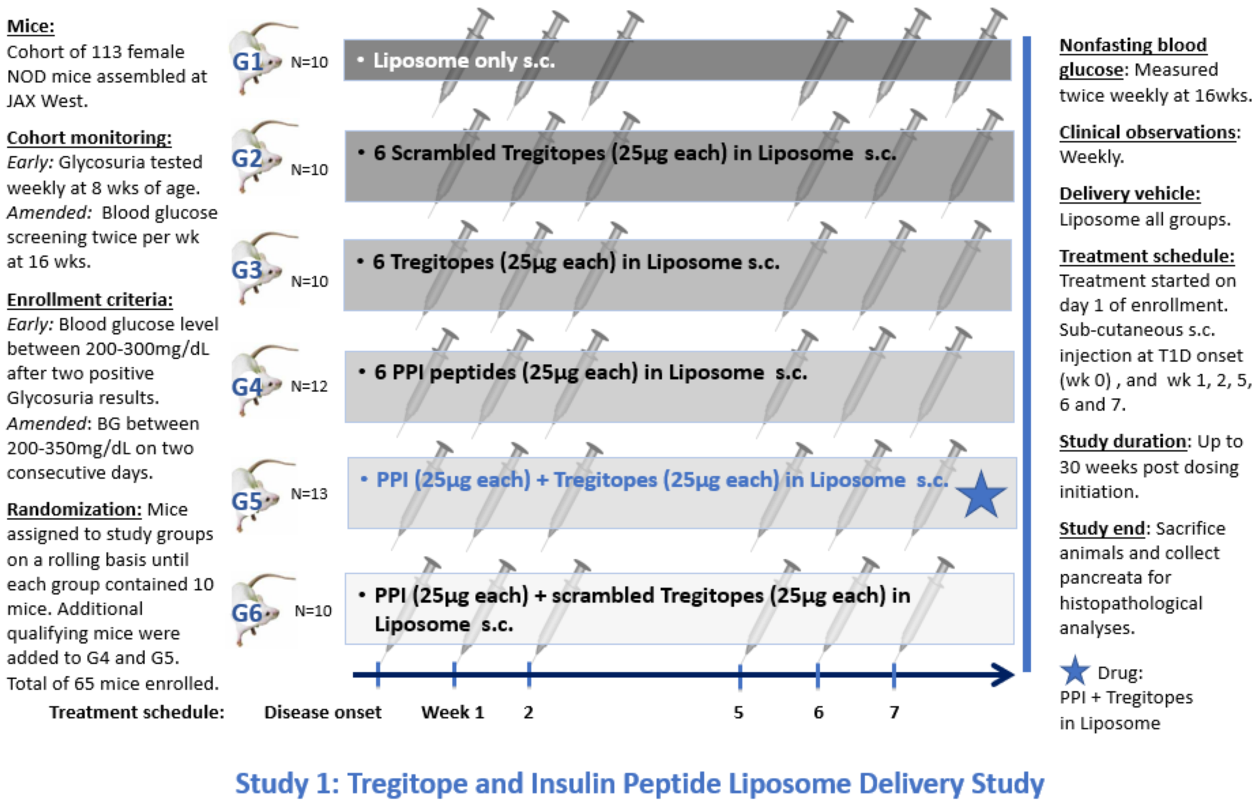

Supplemental Figure S1A: Tregitope in Liposome Study Design. One-hundred and thirteen NOD/ShiltJ (JAX# 1976) female mice were monitored for onset of diabetes by weekly urine testing for glycosuria from 8 weeks of age. After two consecutive positive glycosuria tests, a blood glucose (BG) measurement was

performed to confirm a BG levels between 200-300mg/dL for study entry. Prior to initiating the formal study, it was noted that many mice progressed rapidly beyond 300mg/dL at the second glycosuria test and were not able to be randomized. Therefore, the recruitment protocol was amended to perform two consecutive BG to confirm diabetes onset and to check that the BG level remained between 200-350 mg/dL at enrollment.

The initial number of mice in each group is indicated to the right of each colored bar. The goal was to include at least 10 mice per group. A total of 113 mice were initially enrolled, but many were excluded after the first BG due to rapid increase of the level at the second check (see recruitment details and protocol modification above). Since some mice that qualified over the target number of 60 became available, a total of five extra mice were split between two study groups (G4 and G5) and were also included in the final data analysis.

Each liposome study group G1-G6 received a total of six treatments (on Days 0, 7, 14, 35, 42 and 49) via subcutaneous (s.c.) injection with combinations of PBS (vehicle control with identical DMSO concentration as the peptides), PPI peptides and/or Tregitopes or scrambled Tregitope peptides (control). In all arms of the study, liposomes were formulated with one or combined (e.g. PPI and Tregitopes) test articles. For example, in the proposed T1D-ASATI "drug treatment" arm (starred), PPI peptides were formulated with the Tregitopes and co-administered in the same liposomes.

S1B. Pilot toxicity study

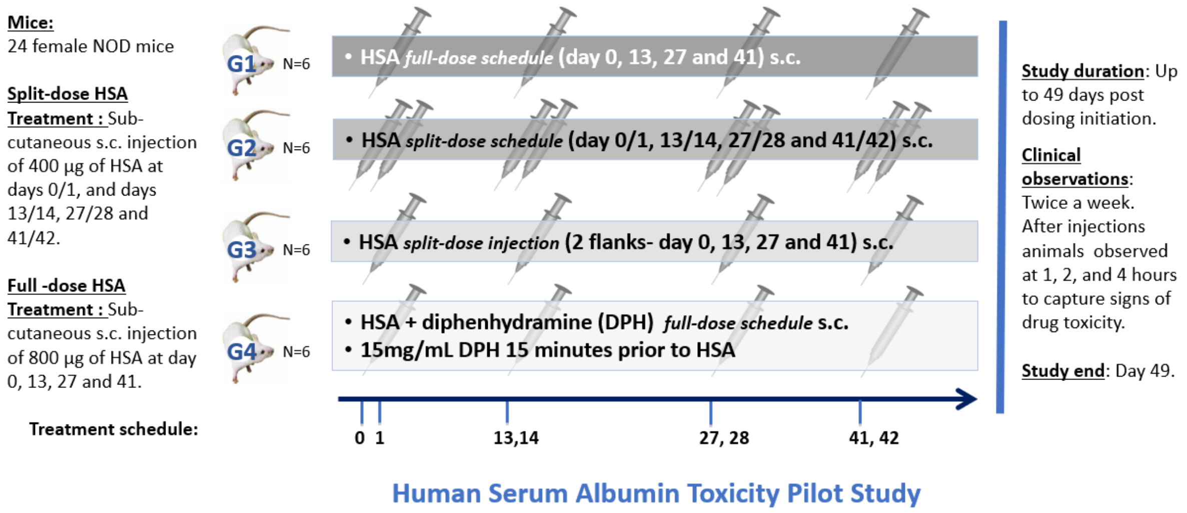

Legend for Supplemental Figure S1B: Due to well established concern about adverse reactions to human serum albumin in NOD mice, a pilot study was performed to determine the optimal dosing approach for the proposed HSA-Tregitope versus control group (HSA without Tregitope) studies. Four groups of mice (N=6 in each arm) were included in the human serum albumin pilot study. **Group 1** mice received HSA as a full

dose (800 µg), **Group 2** received HSA as a split dose one day apart (400 µg); **Group 3** received a split dose injection of the HSA in left and right flanks on the same day (2 x 400 µg), and **Group 4** received HSA as a full dose (800 µg) but were pretreated with diphenhydramine, an antihistamine, 15 minutes prior to injection. Deaths were recorded in all groups. These deaths only occurred only on the second or later dose of therapy. Mice who died developed illness (lethargy, prostration) within 1-2 hours of dosing. Two thirds, or 67% of mice in the HSA (full dose) arm died soon after the second dose of HSA.

Antihistamine pre-treatment reduced mortality (from 67% to 17%), however, all mice developed antihistamine injection site reactions, characterized by hair loss and skin irritation. Split HSA dose by location (administered on the same day) did not reduce mortality (67%). Split dose by time (two doses of HSA, one day apart) led to the most favorable outcome for HSA administered without concurrent Tregitope, with good tolerance and reduced mortality (one death, third administration, 17%). Based on these results, the split dose by time was selected for both HSA (control treatment) and HSA-Tregitope fusion in the planned HSA-Tregitope fusion study.

S1C. HSA-Tregitope Fusion Study Design

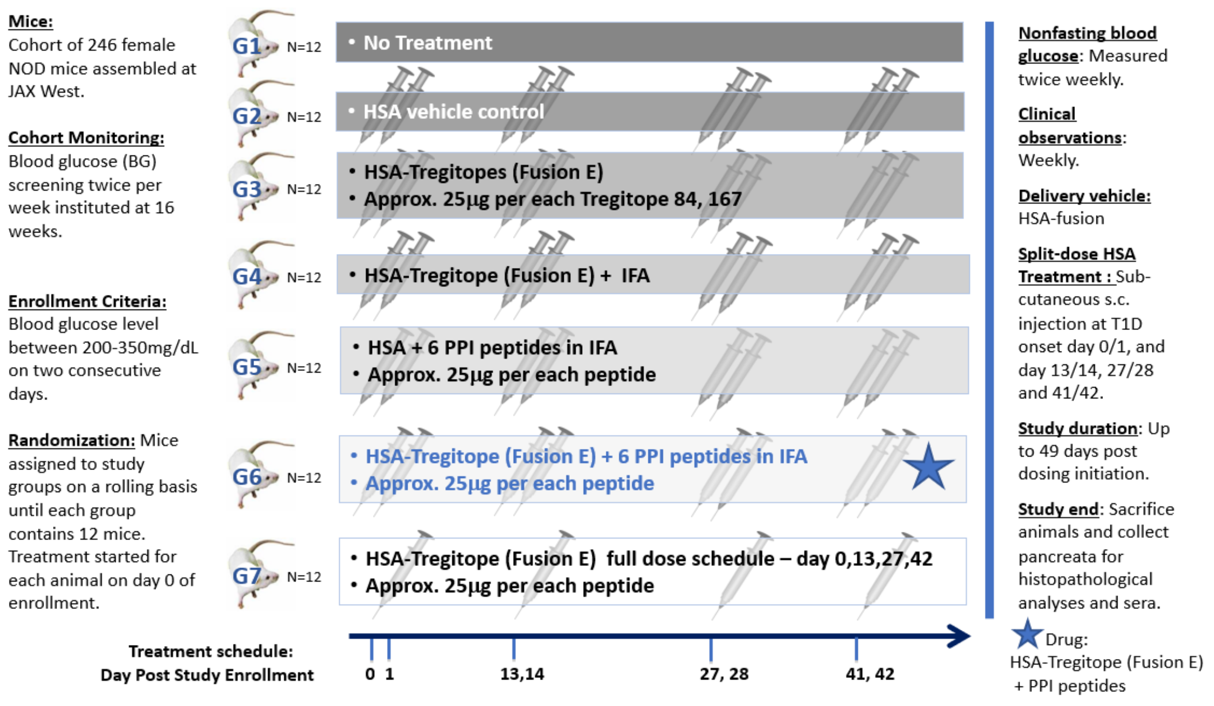

Study 2: HSA-Tregitope Fusion Treatment Study

Legend for Supplemental Figure S1C: Beginning at 16 weeks of age, a total of 246 NOD/ShiltJ (JAX# 1976) female mice were monitored for enrollment into the albumin-Tregitope fusion study. BG monitoring was performed twice weekly using a hand-held glucometer. Mice that had BG values between 200-350

mg/dl were retested as early as possible on the following day and those with a second confirmed BG level between 200-350mg/dl were enrolled into the study at the time of the second testing.

Mice were assigned to seven study groups on a rolling basis until each group contained 12 mice. Mice in groups 2-6 received split dosing of the treatments (Days 0/1, 13/14, 27/28, and 41/42) via subcutaneous injection; mice in group 1 received no treatment and mice in group 7 received single dose HSA-Tregitope treatment (without PPI co-treatment) on days 0, 13, 27 and 41. For those arms receiving peptides (PPI or Tregitopes) approximately 25 ug per peptide was used. Each mouse remained in the study for 49 days after the initial dose of treatment. Clinical observations and body weight measurements were performed on a weekly basis. For injections that occurred after study Day 0, cage side observations were carried out at 1, 2 and 4 hours post injection to identify any signs of toxicity due to the treatments. Mice that appeared moribund, as demonstrated by hunched posture and unkempt fur or who exhibited weight loss exceeding 20%, were euthanized.

Supplemental Figure S2. Complete, uncropped gel of the structure and characterization of HSA-Fusions A-E (Tregitope) protein, as produced in yeast (Novozymes)

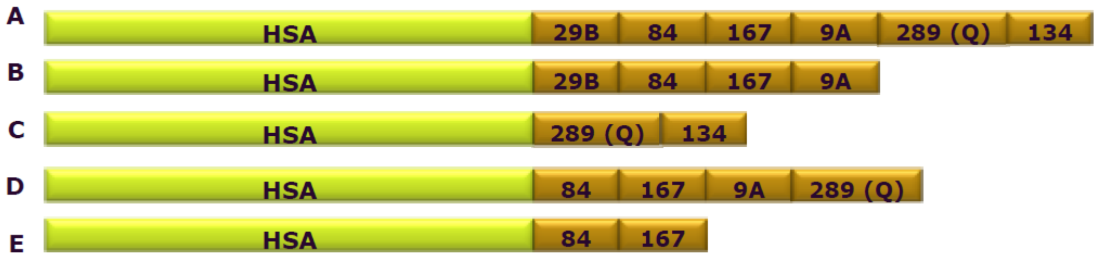

	a.a.	MW (kDa)
Fusion A	707	79.7
Fusion B	662	74.4
Fusion C	630	71.8
Fusion D	668	75.2
Fusion E	626	70.6
HSA	583	66.9

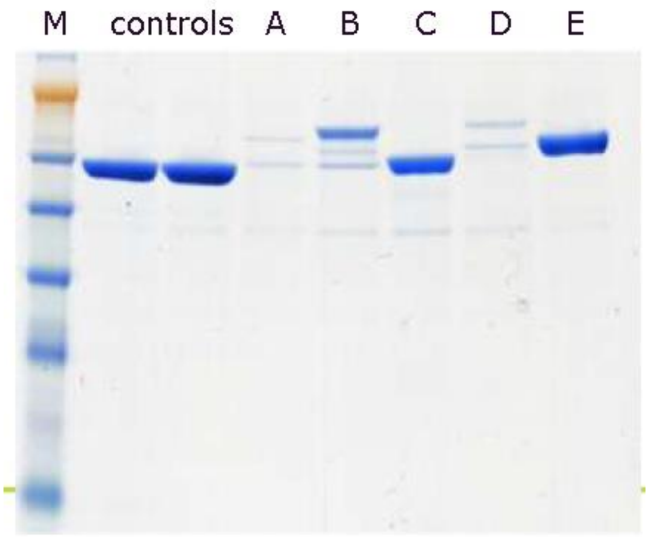

Legend for Supplemental Figure S2: Five albumin-Tregitope fusions were produced by Novozymes (Nottingham, UK). Each human albumin fusion contained an N-terminal string consisting of two, four or six Tregitope strings fused to the C-terminus of human serum albumin. See Blue^{TM} protein standard markers that are used in gels of this type. Controls are recombinant human albumin from the same (yeast) source.

Supplemental Figure S3. Blood glucose heat map for liposome delivery study

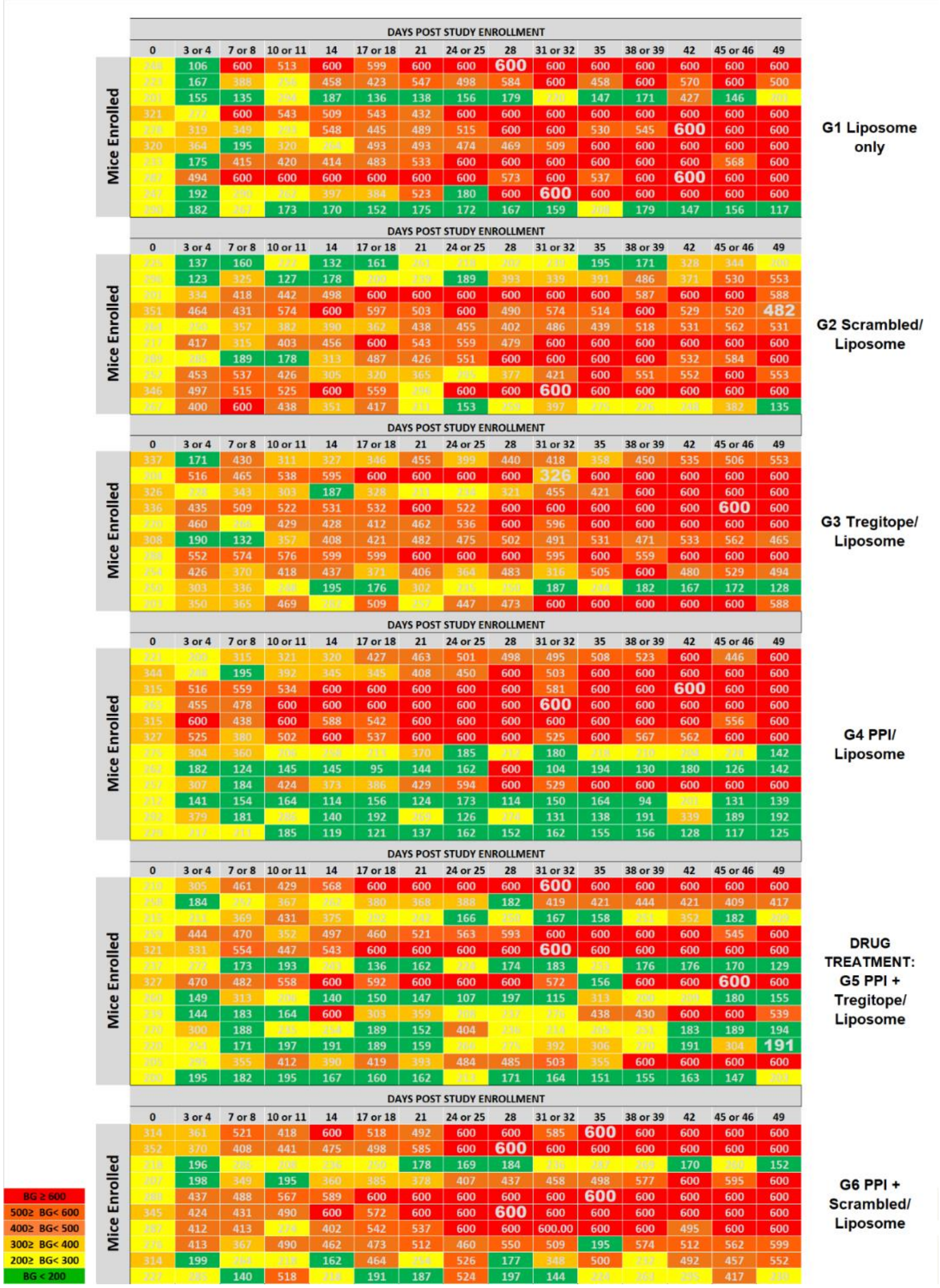

Supplemental Figure S3. Heat map of blood glucose (BG) levels over time for individual mice in the liposome study. BG measurements were made twice per week post enrollment. Each row represents the BG data over time from an individual mouse. Parameters for colors are: <200 (green), 200-300 (yellow), 300-400 (gold), 400-500 (light orange), 500-599 (dark orange), 600 or greater (red). Heat maps are shown for all groups, Groups (G1-G4) and G6 are control groups, and G5 is the PPI + Tregitope "T1D ASATI" group. Bolded BG values indicate when mice were removed from the study for death or weight loss.

Supplemental Figure S4. Blood Glucose Heat map for HSA-Tregitope treatment study

G1 No Treatment

DAYS POST STUDY ENROLLMENT

Mice Enrolled	-1	0	3	7	10	14	17	21	24	28	31	35	38	42	45	49
	274	217	256	192	113	192	260	169	150	227	148	193	210	174	214	461
	245	278	411	556	464	554	530	600	600	600	600	516	600	600	600	600
	328	264	183	203	311	414	355	559	529	409	520	519	494	600	505	480
	265	280	371	220	207	155	187	179	166	167	118	133	131	129	152	137
	313	211	454	373	492	537	599	600	600	600	549	600	573	600	600	600
	286	225	245	266	273	240	211	142	165	201	138	137	209	230	205	302
	328	244	138	505	524	500	540	600	502	497	600	435	423	600	600	600
	216	201	154	187	330	390	480	499	561	600	600	600	377	518	600	600
	321	262	403	499	500	600	600	600	455	600	600	600	600	600	600	600
	201	295	233	340	406	392	297	452	600	583	600	600	600	600	600	600
	202	312	135	327	214	296	369	373	455	451	516	600	600	600	527	600
	202	323	394	349	382	466	283	600	600	549	343	518	442	454	510	600

G2 HSA only

DAYS POST STUDY ENROLLMENT

Mice Enrolled	-1	0	3	7	10	14	17	21	24	28	31	35	38	42	45	49
	208	275	115	132	98	105	103	157	104	118	105	116	97	93	94	106
	316	327	448	432	600	558	555	600	600	419	600	600	600	600	600	600
	314	342	558	600	600	600	600	600	549	480	600	600	600	600	600	600
	344	329	401	367	453	507	600	600	600	600	600	600	600	600	600	600
	342	324	600	600	600	593	525	600	600	600	600	600	600	600	600	600
	205	296	343	413	443	384	320	521	510	137	600	600	600	600	600	600
	235	332	346	184	185	188	564	600	434	573	600	474	600	600	600	600
	265	337	211	268	246	284	233	219	167	600	600	600	600	600	600	600
	200	249	131	402	492	551	572	600	481	600	600	600	600	600	600	600
	299	231	146	276	338	223	319	266	468	438	328	278	386	223	297	226
	202	343	367	557	457	556	544	589	410	374	600	600	600	600	600	600
	324	214	271	234	355	288	465	206	162	184	159	326	184	283	282	241

G3 HSA-Fusion E only

DAYS POST STUDY ENROLLMENT

Mice Enrolled	-1	0	3	7	10	14	17	21	24	28	31	35	38	42	45	49
	305	249	387	544	419	484	530	600	600	600	450	600	600	600	600	600
	313	341	287	520	600	543	600	600	600	600	565	521	600	600	600	600
	268	202	163	160	156	167	139	125	169	194	240	194	355	313	313	323
	217	213	135	376	349	325	535	355	600	530	586	477	600	585	600	476
	221	201	191	201	147	186	132	97	163	203	99	131	141	199	214	180
	299	310	270	237	214	406	242	425	266	557	316	351	182	268	211	206
	258	303	162	210	253	410	376	523	600	556	600	600	600	600	600	600
	318	343	260	335	371	492	448	493	516	585	600	523	600	600	520	600
	231	267	130	102	170	146	115	223	108	158	133	153	143	140	155	174
	220	203	191	170	160	238	150	158	184	170	143	162	246	197	221	195
	221	291	253	170	170	364	299	349	315	407	315	179	201	136	220	157
	285	308	498	540	413	550	569	455	589	600	462	592	600	600	600	600

G4 HSA-Fusion E + peptide vehicle in IFA

DAYS POST STUDY ENROLLMENT

Mice Enrolled	-1	0	3	7	10	14	17	21	24	28	31	35	38	42	45	49
	224	207	331	532	419	486	460	591	486	600	600	600	435	600	600	600
	250	279	289	371	187	233	295	188	208	263	198	147	169	128	169	169
	205	321	334	163	287	326	335	348	369	313	357	336	381	377	334	474
	259	304	589	462	531	483	600	477	498	600	600	600	600	600	600	600
	223	347	200	187	99	136	122	115	134	154	146	203	123	117	154	153
	328	202	195	245	155	148	143	109	181	169	185	148	124	134	203	120
	215	333	429	578	537	600	600	600	474	532	600	600	600	600	600	600
	274	298	245	259	201	171	172	259	181	128	183	220	193	235	264	210
	210	249	429	386	509	498	600	600	600	428	494	487	600	600	600	600
	331	253	267	340	376	410	481	468	537	396	520	450	501	571	575	600
	316	292	407	419	485	439	430	329	276	277	275	172	222	132	170	170
	350	318	424	415	408	512	600	580	519	463	578	598	485	600	600	600

G5 HSA + PPI in IFA

DAYS POST STUDY ENROLLMENT

Mice Enrolled	-1	0	3	7	10	14	17	21	24	28	31	35	38	42	45	49
	283	244	229	194	207	383	357	494	474	600	525	590	600	600	600	600
	214	322	368	388	400	600	492	600	562	600	600	600	600	600	600	600
	232	248	495	437	600	445	600	572	600	380	600	600	600	600	600	600
	234	206	139	127	123	154	124	138	174	206	112	170	118	153	137	150
	228	205	150	127	116	165	145	152	130	212	115	171	96	200	151	114
	264	312	125	175	169	331	292	175	146	145	150	153	158	133	153	139
	252	288	412	291	328	466	552	479	473	506	455	600	389	600	600	600
	223	337	501	465	541	552	555	503	585	600	600	600	600	600	600	600
	303	276	227	237	253	194	182	163	130	128	171	158	111	167	183	149
	263	235	249	274	316	342	300	401	489	449	429	600	448	600	600	499
	279	325	456	546	577	559	549	600	600	600	600	600	600	600	600	600
	223	322	252	333	581	600	600	600	600	600	600	600	600	600	600	600

DRUG TREATMENT: G6 HSA-Fusion E + PPI in IFA

DAYS POST STUDY ENROLLMENT

Mice Enrolled	-1	0	3	7	10	14	17	21	24	28	31	35	38	42	45	49
	208	331	505	532	513	600	523	600	600	600	600	600	600	600	600	600
	278	243	184	182	131	171	168	157	129	129	129	129	129	129	129	129
	324	337	334	363	356	413	580	489	536	600	600	562	600	572	600	600
	337	294	295	461	511	463	374	390	213	245	198	203	130	196	228	239
	235	291	255	263	124	179	139	179	185	210	147	165	138	154	183	125
	230	286	125	155	176	192	177	136	143	114	140	130	139	126	148	157
	274	202	270	207	169	193	211	172	180	164	208	169	179	205	154	139
	246	273	294	146	261	365	209	222	126	211	171	119	187	160	203	123
	285	223	546	600	504	600	549	593	560	491	416	600	600	600	600	600
	355	348	266	310	286	418	264	173	232	257	217	150	167	146	204	131
	218	292	395	257	354	313	462	457	600	600	514	429	344	379	324	428
	336	292	340	324	365	494	445	576	600	600	376	437	600	600	600	600

G7 HSA-Fusion E only (full-dose)

DAYS POST STUDY ENROLLMENT

Mice Enrolled	-1	0	3	7	10	14	17	21	24	28	31	35	38	42	45	49
	221	236	250	144	153	223	161	124	168	197	210	418	539	490	600	600
	301	331	332	476	470	600	600	600	600	600	600	600	600	600	600	600
	267	230	319	427	405	527	518	600	600	600	600	595	408	510	600	600
	293	203	257	378	348	333	280	454	416	491	368	409	579	548	500	483
	201	274	137	155	126	175	141	184	158	142	118	163	115	202	131	113
	202	219	154	214	158	192	163	190	137	197	152	179	138	204	185	118
	217	216	151	155	137	184	244	229	239	135	174	196	230	393	342	363
	287	299	192	365	325	493	600	580	527	498	600	600	600	600	600	600
	306	345	442	402	570	561	541	521	600	600	600	600	600	600	600	600
	230	342	363	433	424	264	287	324	287	466	310	355	431	397	484	483
	234	303	402	442	402	544	475	600	600	600	600	600	600	600	600	600
	202	296	371	386	570	499	520	486	600	600	600	600	600	600	600	600

BG ≥ 600
500≥ BG< 600
400≥ BG< 500
300≥ BG< 400
200≥ BG< 300
BG < 200

Supplemental Figure S4. Heatmap of blood glucose (BG) levels over time for individual mice in the Tregitope-HSA fusion study. BG measurements were made twice per week post enrollment. Each row represents the BG data over time from each individual mouse. Parameters for colors are the same as above. Heat maps are shown for all groups, Groups (G1-G5) are control groups, G6 is the HSA-Fusion E + PPI/IFA therapeutic "drug treatment" group. Mice enrolled in Groups (G1-G6) were given HSA or HSA-Fusion E as a split-dose over 2 days, due to the toxicity observed in pilot study with the HSA as a single dose. Only group G7 received full dose (not split) HSA-Fusion E to demonstrate the lack of toxicity of Tregitope-HSA fusions and their potential for tolerizing against human serum albumin itself in these hyper-reactive mice. Bolded BG values in the heat maps indicate when individual mice died or were removed from the study for weight loss. Mice which expired prior to the 49-day study time course were assigned the maximum BG level (600) in the heat map for subsequent timepoints occurring after death.

Supplemental Figure S5. Comparison of Mean Blood Glucose over time for all groups in the Liposome and HSA-Fusion studies

S5A. Tregitopes and PPI (Preproinsulin Peptide) Liposome Study

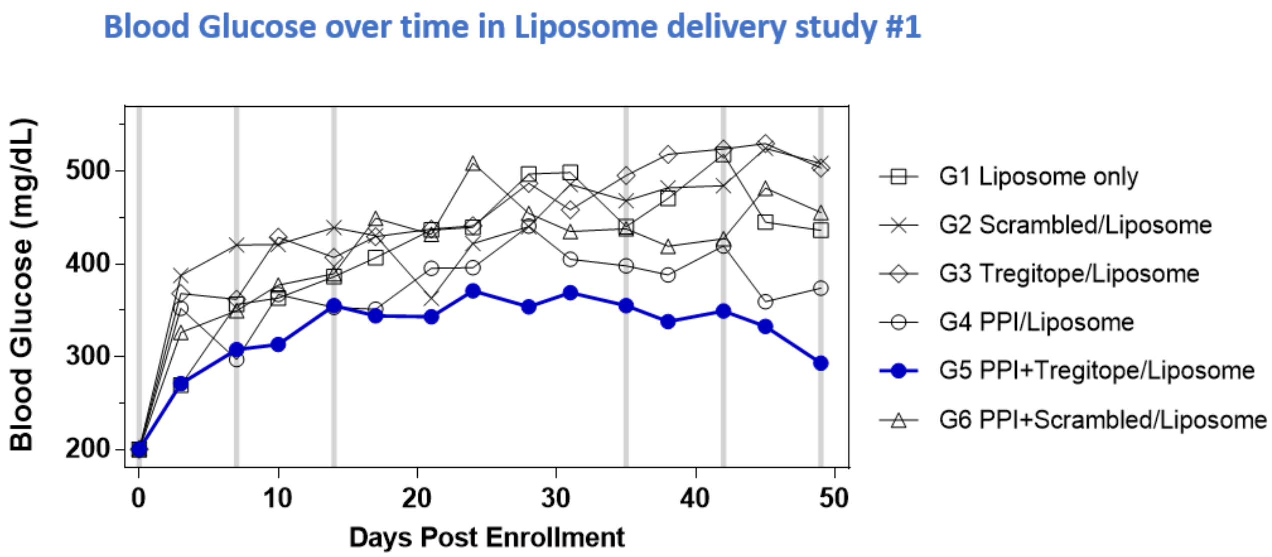

S5B. HSA-Tregitope Fusion and PPI Study

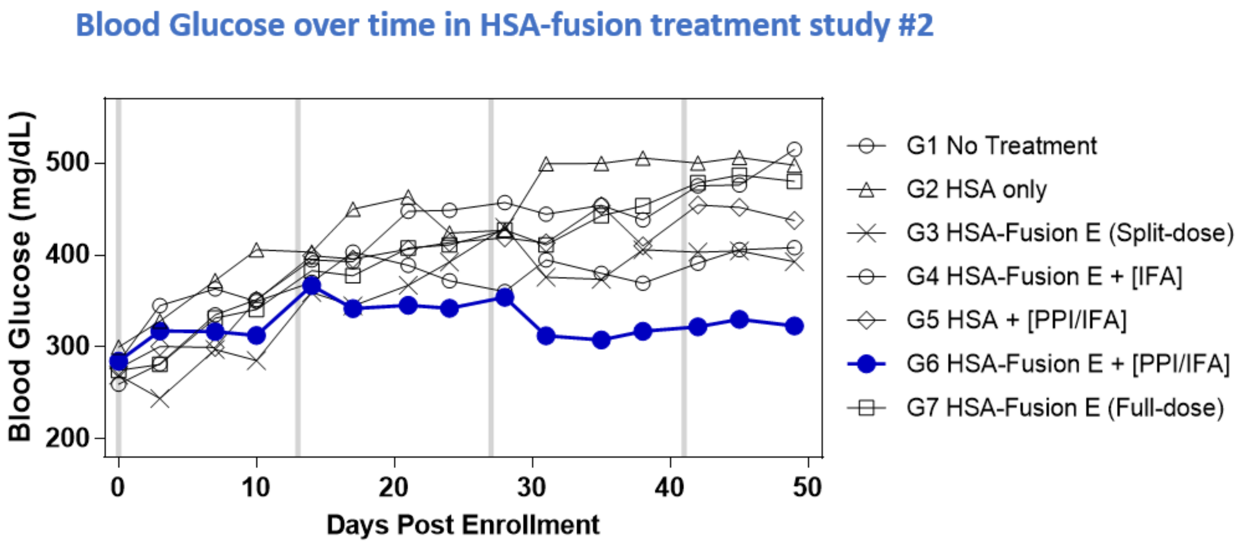

Supplemental Figure S5: Lines representing mean blood glucose for each study group are provided for side-by-side comparisons. Liposome study data is shown in Figure S5A and Tregitope-HSA fusion data is shown in S5B. Mice treated with combination therapy (Tregitope ASATI, dark blue line and solid circles), such as Tregitopes with the target antigen (PPI) in the

same vehicle (S5A) or contemporaneously administered (S5B), fared better than control mice, in both studies. Statistical comparisons between treatment (T1D-ASATI) and individual control groups are provided in Figures 2 and 6 in the main article.

Supplemental Figure S6. Blood Glucose Control Comparison

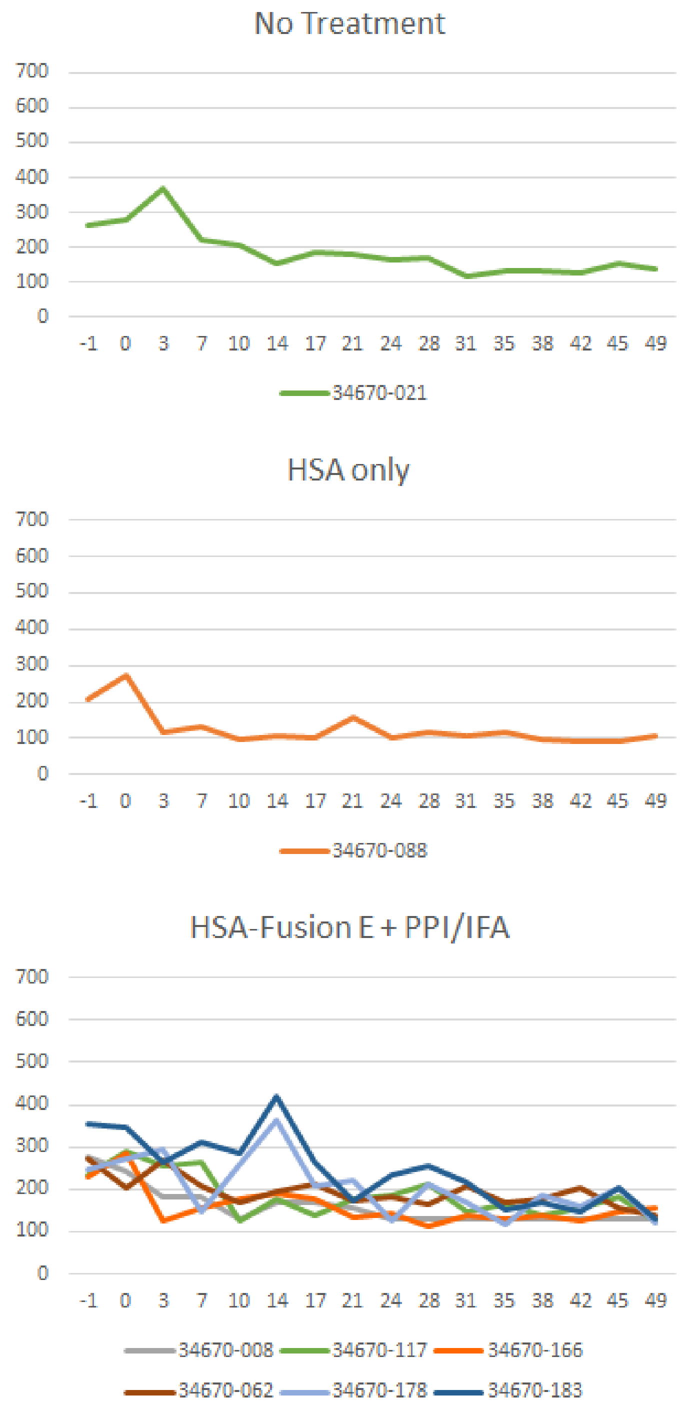

Supplemental Figure S6: HSA Tregitope fusion treatment study showing Blood Glucose over time for individual mice that had BG<200 at the end of the study (Day 49)

Blood glucose (BG) trajectories are shown for mice who controlled their diabetes for three study arms of the HSA-Tregitope fusion study. Mice who did not control their diabetes (BG≥200) are not included in these figures. More mice in the HSA-Fusion E arm were able to control BG even after having developed diabetes. Lower BG was not related to diabetes-associated anorexia (weights remained stable). All mice resolving their diabetes gained weight or had minimal weigh loss (No treatment: -1.7%; HSA only: +7.4%; HSA-Fusion E + PPI/IFA: -1.7% to +5.85%).

Supplemental Figure S7. Insulitis Score vs. Q-Twist for the HSA-Fusion Study

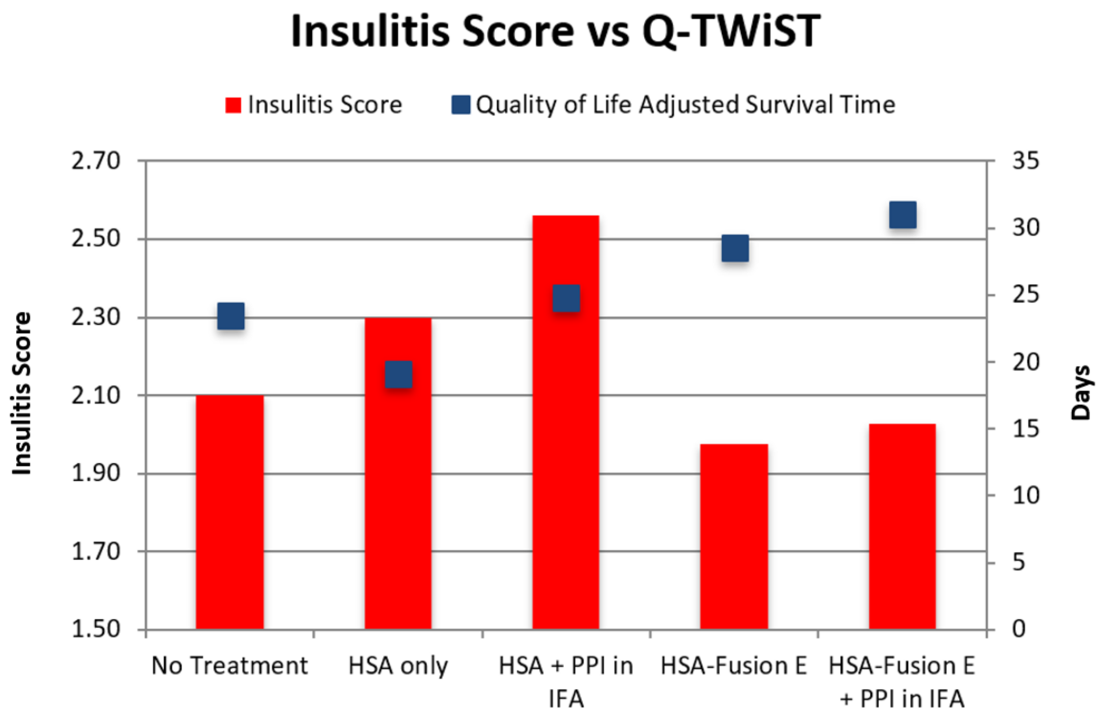

Supplemental Figure S7. Pancreatic tissue was obtained from a subset of mice (n=22) from the HSA-Tregitope fusion study and submitted for pathological analysis. Three mice were examined from the G1 (No Treatment) and G2 (HSA) groups. Four mice were evaluated from the G3 HSA-Fusion 4 mice, five mice were included from the G4 group, HSA + PPI, and six mice were included from the G6 HSA-Fusion E + PPI Group. Mice were euthanized and pancreases were dissected free and fixed in 10% neutral-buffered formalin. Each pancreas was sectioned into 3 levels 100 µm apart. Granulated cells were stained with aldehyde fuchsin (AF) and leukocytes were stained with a hematoxylin and eosin counterstain.

Islets were individually assessed microscopically and score for insulitis. Previously described [35, 36] criteria were used to establish scoring criteria as follows: 0, no lesions; 1, peri-insular leukocytic aggregates; 2, <25% islet destruction; 3, >25% islet destruction; 4, complete islet destruction. An insulitis score for each mouse was obtained by dividing the total score for each pancreas by the total number of islets examined. Microscopic examination was performed by expert technicians who were blinded to treatment group. Group data are presented as mean insulitis score. Slide images were obtained with an Olympus DP72 microscope digital camera. The average insulitis score for each group is plotted along with the Q-Twist scores calculated for each group .

The average insulitis scores per group were plotted against the Q-TWiST scores for quality of life (see Figure 7, higher Q-TWiST indicates higher cumulative duration of time in a lower blood glucose group). Although this study was not powered to determine significant differences in insulitis scores, a trend is observed that mice treated with HSA-Fusion E + PPI (the 'drug treatment arm') had lower scores compared to the control groups. Lower insulitis scores were associated with higher Q-TWiST scores.

---

[35] Serreze, D. V. et al. B lymphocytes are critical antigen-presenting cell for the initiation of T cell-mediated autoimmune diabetes in nonobese diabetic mice. *J. Immunol*. **161,** 3912–3918 (1998).
[36] Takaki, T. et al. HLA-A*0201-restricted T cells from humanized NOD mice recognize autoantigens of potential clinical relevance to type 1 diabetes. *J. Immunol.* **176,** 3257–3265 (2006).
